# Protective anti-fibrotic effect of liraglutide and Pirfenidone combination therapy on liver fibrosis in rats: effects on autophagy and NLRP3 inflammasome

**DOI:** 10.1186/s12876-025-04545-z

**Published:** 2025-12-18

**Authors:** Zeynab Yousefi, Rayan Rajabi, Saeed Karima, Mitra Nourbakhsh, Abbas Sahebghadam Lotfi

**Affiliations:** 1 Basic and Molecular Epidemiology of Gastrointestinal Disorders Research Center, Research institute for Gastroenterology and Liver Diseases, Shahid Beheshti University of Medical Sciences, Tehran, Iran; 2 Metabolic Disorders Research Center, Endocrinology and Metabolism Molecular-Cellular Sciences Institute, Tehran University of Medical Sciences, Tehran, Iran; 3 Oncopathology Research Center, Iran University of Medical Sciences, Tehran, Iran; 4 Department of Clinical Biochemistry, School of Medicine, Shahid Beheshti University of Medical Sciences (SBMU), Tehran, Iran; 5 Department of Clinical Biochemistry, School of Medicine, Iran University of Medical Sciences, Tehran, 1449614535 Iran; 6 Department of Clinical Biochemistry, Faculty of Medical Sciences, Tarbiat Modares University, Jalal Ale Ahmad, Nasr, P.O. Box: 14115-111, Tehran, Iran; 7 Iran University of Medical Sciences, Hemmat Highway, P.O. Box: 1419614535, Tehran, Iran

**Keywords:** Autophagy, Inflammasome, Liraglutide, Liver fibrosis, Liver regeneration pirfenidone

## Abstract

**Background:**

Liver fibrosis is a significant complication of chronic liver diseases. While Pirfenidone (PFD) and Liraglutide (LIR) have shown promise individually in treating fibrosis, their combined effect on autophagy and NLRP3 inflammasome pathways remains largely unexplored.

**Methods and results:**

This study investigated the protective effects of combined LIR and PFD therapy on autophagy and NLRP3 inflammasome, fifty male Wistar rats were divided into five groups: Sham, BDL, BDL + PFD (200 mg/kg), BDL + LIR (200 µg/kg), and BDL + PFD + LIR combination. Following 20 days of treatment, liver tissues were analyzed for histological and immunohistochemical (IHC) changes, biochemical parameters, and molecular markers of fibrosis, autophagy, and inflammasome activation. The combination therapy significantly reduced serum liver injury markers (ALT, AST, ALP), decreased ECM deposition, and improved histological parameters compared to monotherapy. Combined treatment effectively suppressed inflammatory markers (NF-κB, TNF-α) while increasing anti-inflammatory IL-10. Furthermore, the combination therapy modulated autophagy markers (Beclin 1), cathepsin B, and reduced NLRP3 inflammasome activation (NLRP3, Caspase 1, IL-1β, IL-18) more effectively than either drug alone. IHC staining of Ki-67 and HepPar-1 showed that combination therapy enhanced expression of proliferative and differentiation markers.

**Conclusions:**

PFD and LIR combination therapy demonstrates superior therapeutic efficacy in treating BDL-induced LF through enhanced liver regeneration through enhanced expression of proliferative and differentiation markers and modulation of autophagy and NLRP3 inflammasome pathways, indicating that the combination of PFD and LIR represents a promising therapeutic strategy for LF.

**Supplementary Information:**

The online version contains supplementary material available at 10.1186/s12876-025-04545-z.

## Introduction

Cholestatic liver disease progressively leads to fibrosis, cirrhosis, and ultimately liver failure [1]. Hepatic tissue experiences several structural and metabolic alterations in cases of extensive liver injury, such as bile duct ligation (BDL)-induced liver fibrosis (LF), which represents a complex pathophysiological process. Activation of hepatic stellate cells (HSCs) leads to the creation of fibrous tissue and an increase in collagen production in the extracellular matrix (ECM) [2]. By releasing cytokines that cause inflammation and forming fibroblasts in response to different traumas, hepatocytes contribute to the pathophysiology of LF [3]. Chronic inflammation plays a central role in liver fibrosis by driving hepatic stellate cell activation, promoting their transition into collagen-producing myofibroblasts and accelerating extracellular matrix accumulation, thereby contributing directly to fibrosis progression [4, 5].

Autophagy is a lysosome-mediated and evolutionarily conserved cellular mechanism that breaks down damaged organelles, protein aggregates, and other macromolecules in the cytoplasm. It controls cell death in both healthy and diseased settings [6, 7]. Dysregulated autophagy has been implicated in various liver disorders, including hepatic fibrosis [8]. The role of autophagy in LF is complex and context-dependent, with evidence showing it can both promote and inhibit fibrosis. In the present study, we aimed to explore its specific role and modulation by therapy [9, 10]. Growing evidence suggests that autophagy regulates the NLRP3 inflammasome signaling pathway during liver fibrosis [11].

Inflammation plays a pivotal role in the activation of HSCs, which are the major effector cells in liver fibrogenesis. Persistent liver injury leads to the recruitment of inflammatory cells and the release of cytokines such as TGF-β, IL-1β, and TNF-α. These mediators promote the transdifferentiation of quiescent HSCs into activated myofibroblast-like cells that produce excessive ECM proteins, including type I collagen. Consequently, the inflammatory microenvironment not only initiates but also sustains HSC activation and fibrosis progression. Targeting inflammation is, therefore, a crucial strategy to prevent or reverse liver fibrosis [12–14].

One of the NOD-like receptors (NLRs) involved in the innate immune response is NLRP3 (NOD-like receptor, pyrin domain-containing 3) [15]. The inflammasome, a protein complex formed by NLRP3, triggers caspase-1 activation, which leads to the maturation and release of pro-inflammatory cytokines such as IL-1β and IL-18 [16]. Therefore, in response to pathogen or damage-associated molecular patterns by both microbial and nonmicrobial stimuli, the NLRP3 inflammasome signaling pathway controls a number of host innate immune defense pathways [17, 18]. It has been demonstrated in research on fibrosis (lung, kidney, etc.) that mice lacking NLRP3 exhibit reduced cytokine and interleukin output. Collectively, these findings suggest that NLRP3 is a key regulator of fibrosis [19, 20]. Previous research on LF showed that NLRP3 deficiency reduced inflammation, fibrosis, and liver damage [11].

Pirfenidone (PFD, 5-methyl-1-phenyl-2-(1 H)-pyridone) was authorized via the U.S. Food and Drug Administration in 2014 to treat idiopathic pulmonary fibrosis. By lowering pro-fibrotic cytokines (such as TGF-β1), PFD has shown an anti-fibrotic impact. These cytokines slow the progression of fibrosis through a number of methods. These processes include fibroblast proliferation, collagen deposition, epithelial-mesenchymal transition (EMT), and attenuating inflammatory markers [21–23]. Therefore, there have been continuous attempts to investigate a novel therapeutic approach or an alternate treatment for LF. PFD has not yet been approved for the treatment of human LF; however clinical trials are currently underway [24]. Although its precise molecular target remains unclear, PFD has been shown to prevent HSC activation and fibrosis in rodents [25–28]. Additionally, it was proposed that PFD might target hepatocytes and other types of hepatic cells [29].

Combination drug therapy represents a promising strategy for this high-mortality condition. Since it is difficult to get adequate outcomes with single-drug treatment, multi-drug combination therapies have gained more attention [30]. Because of its capacity to stabilize liver function in hepatic illness, liraglutide (LIR), a glucagon-like peptide-1 receptor (GLP-1R) agonist, may serve as a potential candidate for combination therapy. The primary approved use of LIR, an insulinotrophic hormone, is in the management of type 2 diabetes [31]. Many tissues exhibit high levels of GLP-1 expression, including the liver having the highest levels. Both in healthy and diseased situations, GLP-1R activity is crucial for liver function. According to current data, LIR improves the hepatic histological aspects of NAFLD [32]. Furthermore, it has been demonstrated to have a favorable effect on the cellular autophagic response during liver dysfunction and to have very minimal impacts on inflammation brought on by lipotoxicity. In rats with type 2 diabetes, LIR can trigger the cellular autophagic response [33, 34]. LIR is becoming a more appealing therapy option for LF in light of this body of research. Notably, our previous in vivo findings demonstrated that PFD therapy had strong impacts on the regeneration of LF. Given that PFD primarily targets fibrotic pathways and LIR demonstrates potent anti-inflammatory and autophagy-modulating effects, we hypothesized that their combination would produce a synergistic anti-fibrotic response. Further evidence supporting the enhanced effectiveness of PFD and LIR combination therapy on liver regeneration is therefore valuable. Therefore, we postulated that autophagy might control LF following BDL by controlling the NLRP3 inflammasome. Thus, the purpose of this work was to investigate the hepatoprotective effects of combined LIR and PFD therapy on autophagy and the NLRP3 inflammasome in Wistar rats with BDL-induced LF.

## Materials and methods

### Animal

Fifty adult male Wistar rats (250 ± 50 g) were acquired from the Pasteur Institute of Iran’s Laboratory Animal Center. They were housed under controlled environmental conditions (25 ± 2 °C, 50% humidity) with a 12 h light/dark cycle and adequate ventilation. Animals had free access to tap water and standard rodent chow. They had unlimited access to tap water and a typical diet of rodent pellets. The study was approved by the Ethical Committee of the Endocrinology and Metabolism Research Institute, Tehran University of Medical Sciences (Approval No. IR.TUMS.AEC.1402.137). All animal procedures complied with the institutional guidelines for animal care and handling, in accordance with ARRIVE and Tehran University of Medical Sciences regulations.

### Experimental design

**Fig. 1:**
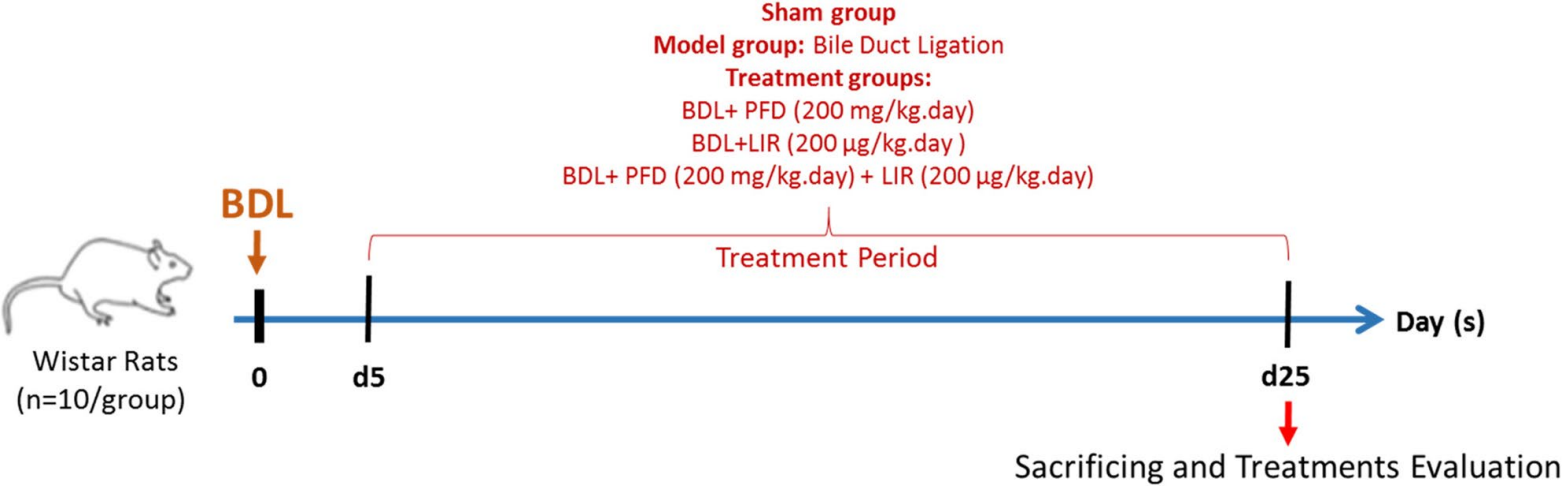
Experimental design and timeline of the study. Liver fibrosis was induced by bile duct ligation (BDL) on day 0. Treatments (PFD, LIR, or both) began 5 days post-BDL and continued daily for 20 days. Animals were euthanized on day 25 for analysis

Before the allocation of the rats, body weight was measured. As presented in Fig. 1, animals were assigned to five groups (*n* = 10 per group). Sham groups, the BDL group (model group), and treatment group including: the BDL + PFD (200 mg/kg body weight) group, the BDL + LIR (200 µg/kg body weight) group, and the BDL + PFD + LIR (PFD = 200 and LIR = 200 µg/kg body weight) group. The sample size was determined based on previous studies [35], and PFD and LIR doses were selected accordingly [35–37]. Prior to biliary obstruction (BDL model induction), rats were given ketamine-xylazine intraperitoneally (i.p.) to induce anesthesia. as previously described [38]. The sham group underwent the same laparotomy without bile duct ligation. After a 5-day recovery period, treatments were administered once daily for 20 days: PFD via oral gavage and LIR via subcutaneous injection. Control animals received an equal volume of physiological saline.

### Collection of tissues and blood sample

At the end of the experiment, rats were euthanized by intraperitoneal injection of a high-dose ketamine/xylazine cocktail, and blood was collected via cardiac puncture. Livers were immediately excised, weighed, and the liver index (liver-to-body weight ratio) was calculated. Blood samples were centrifuged at 3000 rpm for 10 min, and serum was stored at − 80 °C for subsequent biochemical analysis. Liver samples were divided for molecular, histological, immunohistochemical (IHC), and hydroxyproline assays.

## Biochemical and molecular studies

### Biochemical parameter measurement

Alkaline phosphatase (ALP), Aspartate aminotransferase (AST), and Alanine aminotransferase (ALT) levels in serum were assessed using enzymatic methods in compliance with the kit’s instructions (Pars Azmun, Iran).

### Histological analysis

Haematoxylin and eosin (H&E), Masson trichrome, and Sirius Red were used to stain fixed sections of liver samples. An experienced pathologist blindly evaluated and rated each histopathological sample using the fibrosis scoring system [39].

### Immunohistochemical analyses

Liver tissue samples from Wistar rats were subjected to immunohistochemical staining for several markers: HepPar-1 (Anti-Hepatocyte Specific Antigen antibody, mouse monoclonal [OCH1E5], GeneTex, USA, diluted 1:100, Cat. No. GTX73779; validated for rat), α-SMA (Rabbit Anti-ACTA2 Polyclonal antibody, biorbyt, UK, diluted 1:500, Cat. No. orb1533440; validated for rat), and Ki-67 (rabbit monoclonal antibody [SP6], BIOCARE MEDICAL, USA, diluted 1:50, Cat. No. PRM 325 AA, H; validated for rat). Staining was carried out using a Mouse and Rabbit Specific HRP/DAB Detection IHC kit (Abcam, UK, catalog number ab64264). Following deparaffinization and rehydration, sections underwent antigen retrieval and were incubated with primary and secondary antibodies. Negative controls (secondary antibody–only) were included to confirm the absence of nonspecific staining. The stained sections were analyzed microscopically, and positive cells for each marker were counted in 12 consecutive fields to assess expression percentages, which were subsequently evaluated using ImageJ software.

### Hydroxyproline measurement

The hydroxyproline level in liver tissues was measured using commercial hydroxyproline detection kits (Kalazist Life Sciences, Hamedan, Iran) in accordance with the kit’s instructions.

#### RNA isolation and RT-PCR

Total RNA was extracted from frozen liver tissues using TRIzol reagent (Yektatajhiz, Iran). cDNA was synthesized using the RevertAid cDNA Synthesis Kit (Yektatajhiz, Iran). Quantitative real-time PCR was conducted on an ABI StepOne System (Applied Biosystems, USA) using RealQ Plus 2× Master Mix SYBR Green (Amplicon, Denmark). Primer sequences are provided in Supplementary 2. Gene expression was normalized to GAPDH, and relative expression levels were calculated using the 2⁻ΔΔCt method.

### Western blotting

In order to extract proteins, liver tissues were homogenized in lysis buffer. The bicinchoninic acid assay (BCA) kit (Thermo Fisher Scientific, UK) was applied to quantify the protein concentration. The Beclin 1 (Cat. No. sc-48341, Santa Cruz Biotechnology), Cathepsin B (CTSB) (Cat. No. sc-365558, Santa Cruz Biotechnology), NLRP3 (Cat. No. 13158 S, Cell Signalling Technology) and Caspase 1 (Cat. No. sc-56036, Santa Cruz Biotechnology), and GAPDH (Cat. No. sc-32233, Cell Signalling Technology) immunoblotting was carried out (1:1000). The Caspase 1 detected band corresponds primarily to full-length caspase-1 (20 kDa). Secondary antibodies were then applied to the blots (Santa Cruz, USA). Finally, the ECL reagent and Image J software (National Institutes of Health, Bethesda, USA) were used to view the immunoblot.

### Statistical analysis

All data are expressed as the mean ± standard deviation (SD). Statistical analysis was performed using GraphPad Prism version 9 (GraphPad Software Inc., San Diego, CA, USA). Differences among the experimental groups were analyzed using a one-way analysis of variance (ANOVA) followed by Tukey’s multiple comparison post-hoc test to assess intergroup differences. A P value of < 0.05 was considered statistically significant.

## Results

### LIR and PFD alone and their combination alleviate BDL-induced liver fibrosis in rats

**Fig. 2:**
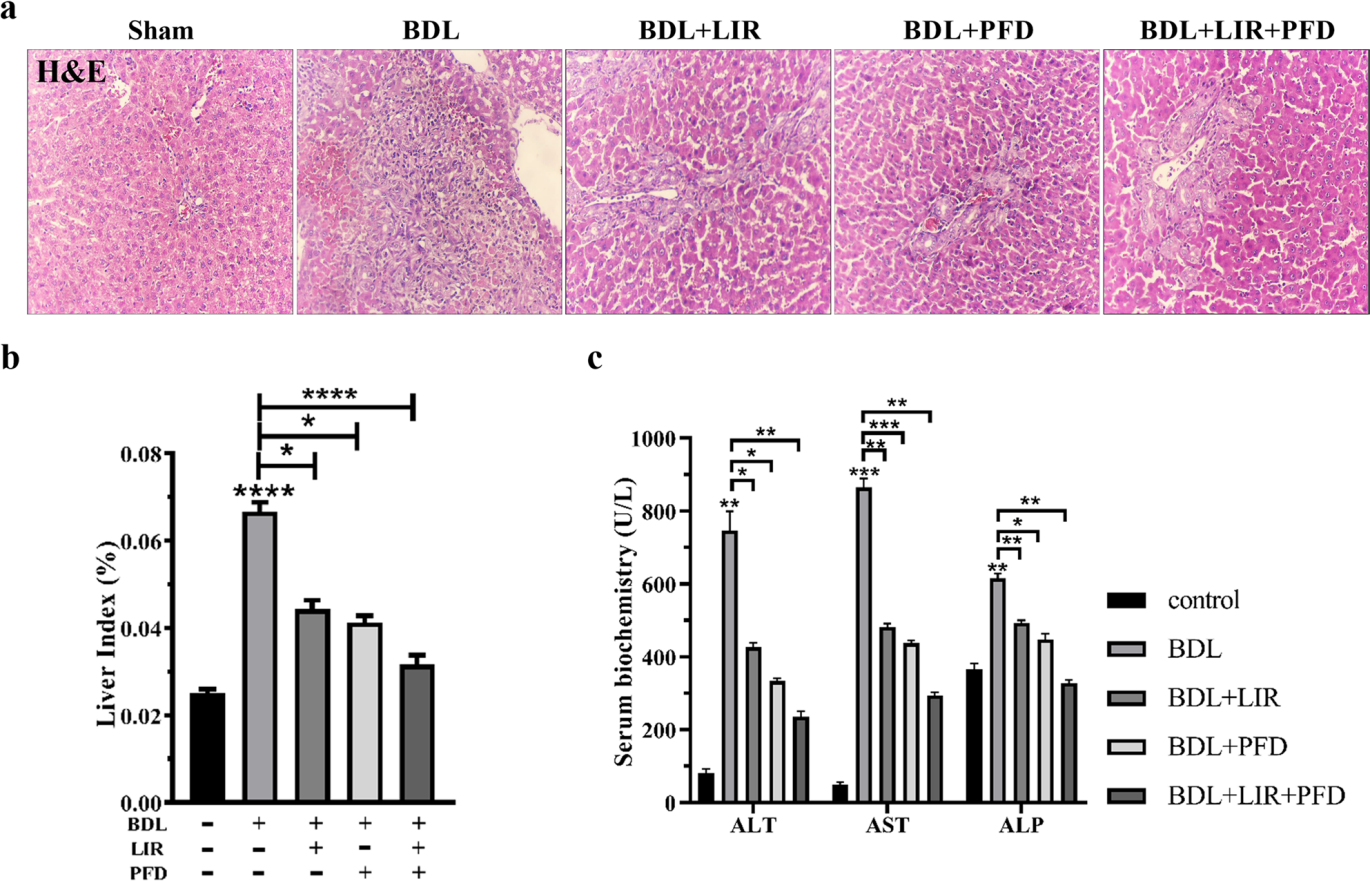
Effect of LIR and PFD alone and combination of LIR + PFD on the BDL-induced rat. **a** H&E staining of different groups for histological examination. Scale bar: 100 μm and magnification: X100. **b** The rat liver/body weight ratio. **c** The serum levels of ALT, AST and ALP. H&E: Hematoxylin and eosin, BDL: Bile duct ligation, PFD: Pirfenidone, LIR: Liraglutide, ALT: Alanine aminotransferase; AST: Aspartate aminotransferase; ALP: alkaline phosphatase. *p < 0.05, ** *p* < 0.01, *** *p* < 0.001, **** *p* < 0.0001

To evaluate the protective effects of LIR, PFD, and their combination against BDL-induced liver fibrosis, histological and biochemical parameters were examined. As expected, rats subjected to BDL exhibited marked pathological alterations compared to the sham group, including hepatocellular necrosis, steatosis, and bridging fibrosis. These changes confirmed successful induction of liver fibrosis. Treatment with LIR or PFD alone noticeably attenuated these histopathological lesions, as shown by reduced fibrotic septa and improved hepatic architecture. Both monotherapies also significantly decreased the elevated liver-to-body weight ratio and serum levels of ALT, AST, and ALP compared with the BDL group. Importantly, the combination therapy (LIR + PFD) provided the greatest hepatoprotective effect. Histological examination showed near-normal liver lobular structure with minimal fibrosis, and biochemical markers were significantly lower than in either monotherapy group. Collectively, these results indicate that while both LIR and PFD individually ameliorate BDL-induced liver injury, their combination exerts a stronger protective effect through additive or synergistic mechanisms (Fig. 2a–c). No significant differences in body weight changes were observed among the experimental groups, suggesting that the observed hepatoprotective effects were not confounded by liraglutide-induced weight loss.

### LIR and PFD alone and their combination alleviate BDL-induced ECM deposition in rats

**Fig. 3:**
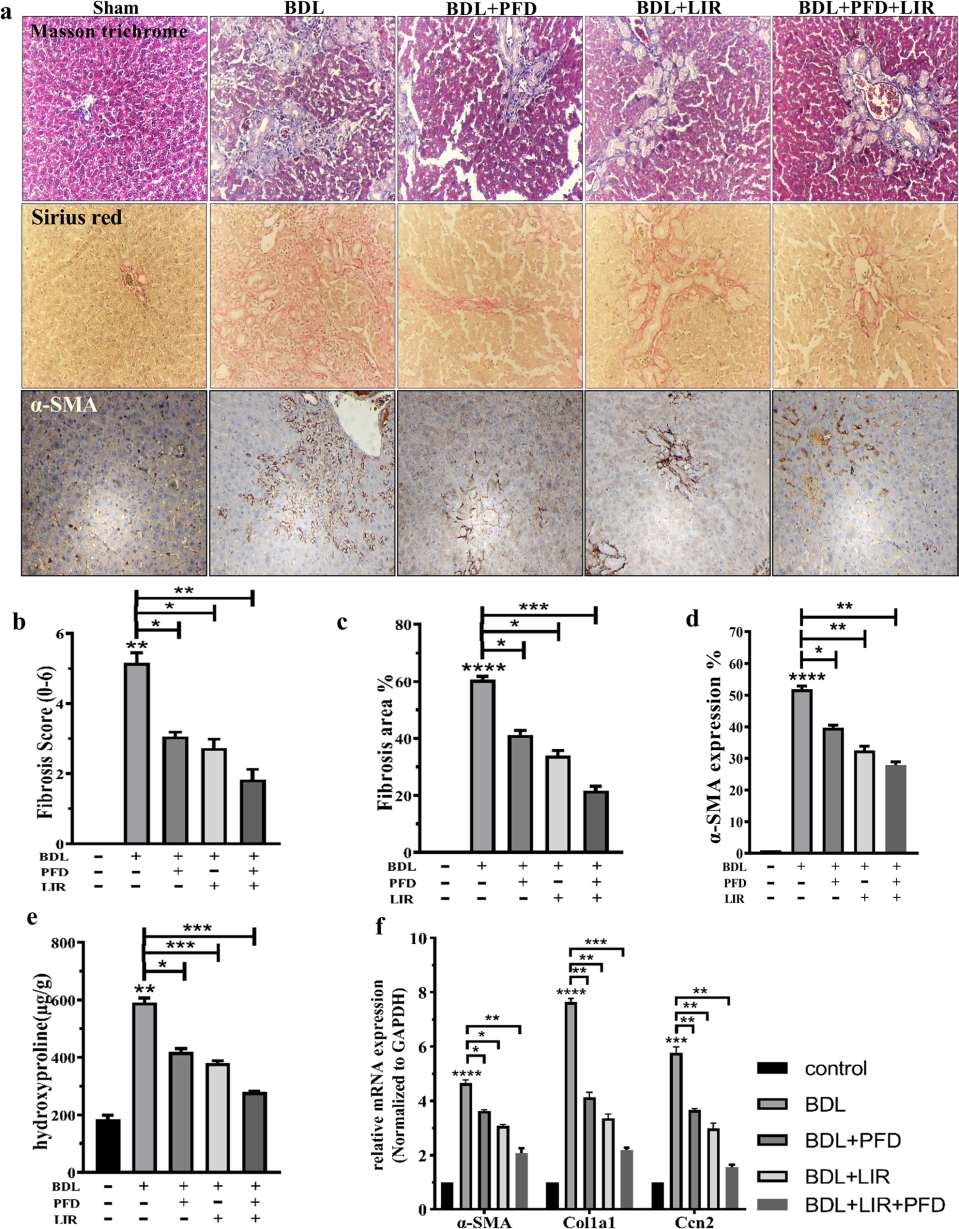
Effects of LIR and PFD alone and LIR + PFD combination on the BDL-induced ECM deposition in rats. **a** Masson trichrome and Sirius Red staining for histological examination and Sections of liver tissue immunostained against α-SMA for different groups. Scale bar: 100 μm and magnification: X100. **b** fibrosis scores and (**c**) fibrosis area; (**d**) The α-SMA expression (%) was recorded by counting the positive cells; (**e**) liver tissue HYP content; and (f) the mRNA expression level of α-SMA, Col1a1, and Ccn2. BDL: Bile duct ligation, PFD: Pirfenidone, LIR: Liraglutide, HYP: Hydroxyproline, α-SMA: Alpha Smooth Muscle Actin, Col1a1: Collagen Type I Alpha 1, Ccn2: Cellular Communication Network Factor 2. *p < 0.05, ** *p* < 0.01, *** *p* < 0.001, **** *p* < 0.0001

Masson’s trichrome and Sirius Red staining revealed normal hepatic architecture in the sham rats, with well-preserved lobular organization and minimal collagen deposition. In contrast, the BDL group showed extensive periductal fibrosis, collagen accumulation, and hepatocellular necrosis, confirming the successful induction of liver fibrosis (Fig. 3a). Treatment with LIR or PFD alone notably reduced collagen deposition and fibrosis around the portal tracts, as evidenced by improved histological structure and decreased fibrosis scores (Fig. 3b–c). Both agents effectively mitigated extracellular matrix (ECM) accumulation compared with the untreated BDL group. Importantly, the LIR + PFD combination therapy demonstrated a more pronounced protective effect than either monotherapy. Collagen fibers were markedly reduced, and liver histology appeared nearly normal. The fibrosis area and score were significantly lower than in the monotherapy groups (Fig. 3b–c), suggesting a synergistic effect. Immunohistochemical staining for α-SMA, a marker of activated hepatic stellate cells, further supported these findings. α-SMA expression was minimal in sham livers but markedly elevated after BDL. Both LIR and PFD reduced α-SMA expression, while the combination therapy resulted in the most substantial decrease (Fig. 3d). Similarly, hydroxyproline (HYP) content, which reflects collagen accumulation, significantly increased in the BDL group. All treatments reduced HYP levels, with the combination therapy showing the greatest reduction (Fig. 3e). At the molecular level, BDL markedly elevated the mRNA expression of α-SMA, Col1a1, and Ccn2, key ECM-related genes (Fig. 3f). All treatments downregulated these genes, and the LIR + PFD combination produced the most substantial suppression, indicating an enhanced anti-fibrotic effect. Collectively, these results demonstrate that while both LIR and PFD attenuate ECM accumulation and fibrosis, their combination exerts superior efficacy in reducing ECM deposition and hepatic stellate cell activation in BDL-induced liver fibrosis.

### Combination therapy of LIR and PFD reduces inflammatory markers in rats with BDL-induced liver fibrosis

**Fig. 4:**
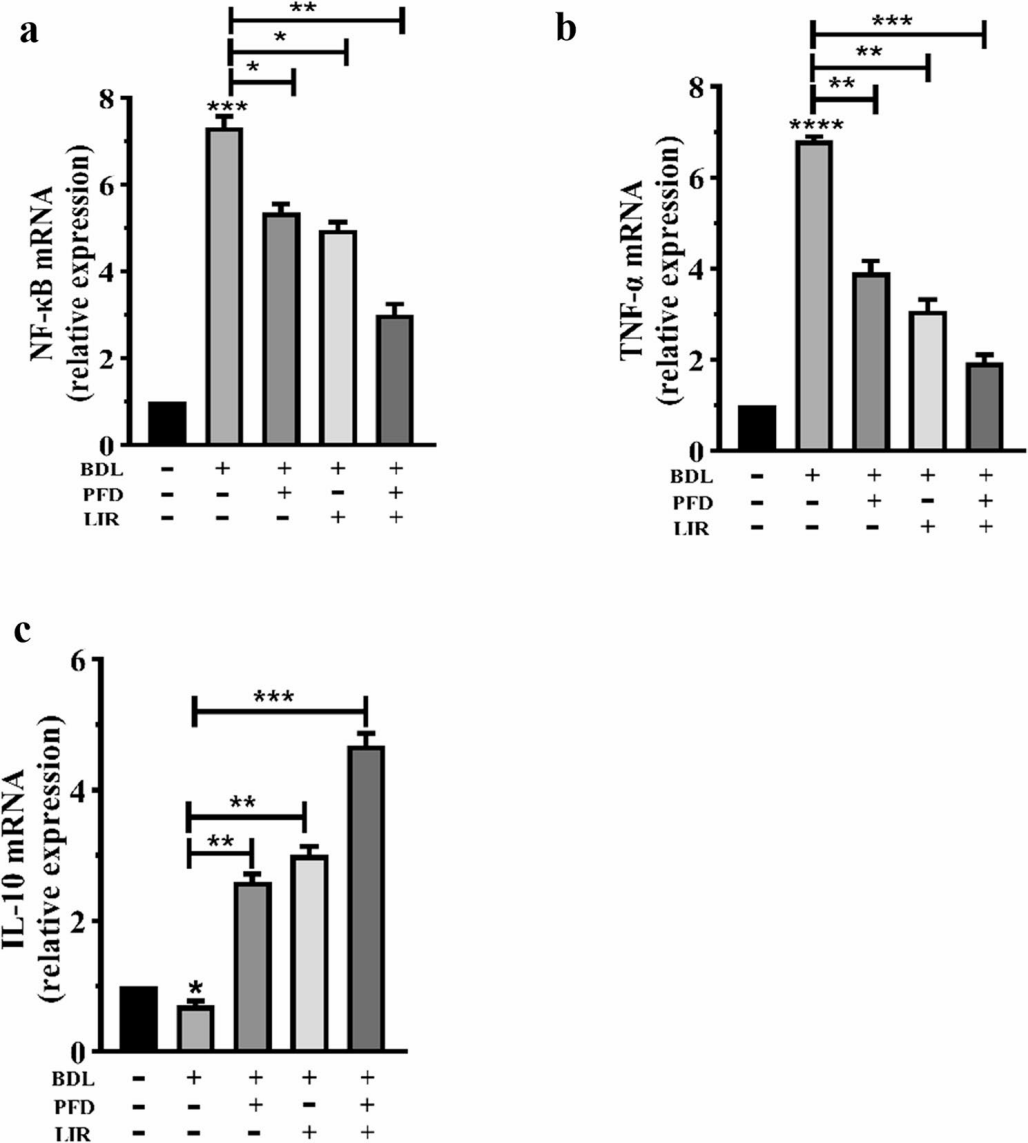
Effects of LIR and PFD alone and LIR + PFD combination on the BDL-induced inflammatory response in rats. The mRNA levels of (**a**) NF-κB, (**b**) TNF-α, and (**c**) IL-10. BDL: Bile duct ligation, PFD: Pirfenidone, LIR: Liraglutide, TNF-α: Tumor Necrosis Factor alpha; NF-κB: Nuclear factor kappa-light-chain-enhancer of activated B, IL-10: Interleukin-10. *p < 0.05, ** *p* < 0.01, *** *p* < 0.001, **** *p* < 0.0001

To evaluate the anti-inflammatory effects of LIR, PFD, and their combination, the mRNA expression of NF-κB, TNF-α, and IL-10 was quantified using RT-PCR. As expected, BDL induction led to a pronounced increase in NF-κB and TNF-α expression, accompanied by a significant reduction in IL-10 mRNA levels, indicating an inflammatory response and loss of immune regulation compared with the sham group (Figs. 4a–c). Treatment with LIR or PFD alone significantly attenuated the pro-inflammatory response, as shown by decreased NF-κB and TNF-α expression and restoration of IL-10 toward normal levels. Importantly, the LIR + PFD combination therapy produced the most marked improvement, with NF-κB and TNF-α levels approaching those of the sham group and IL-10 expression showing the strongest recovery. These findings demonstrate that the combined administration of LIR and PFD more effectively restores the inflammatory balance than either monotherapy, thereby reducing hepatic inflammation in BDL-induced liver fibrosis.

### Combination therapy of LIR and PFD normalized autophagy and NLRP3 inflammasome activation in rats with BDL-induced liver fibrosis

**Fig. 5:**
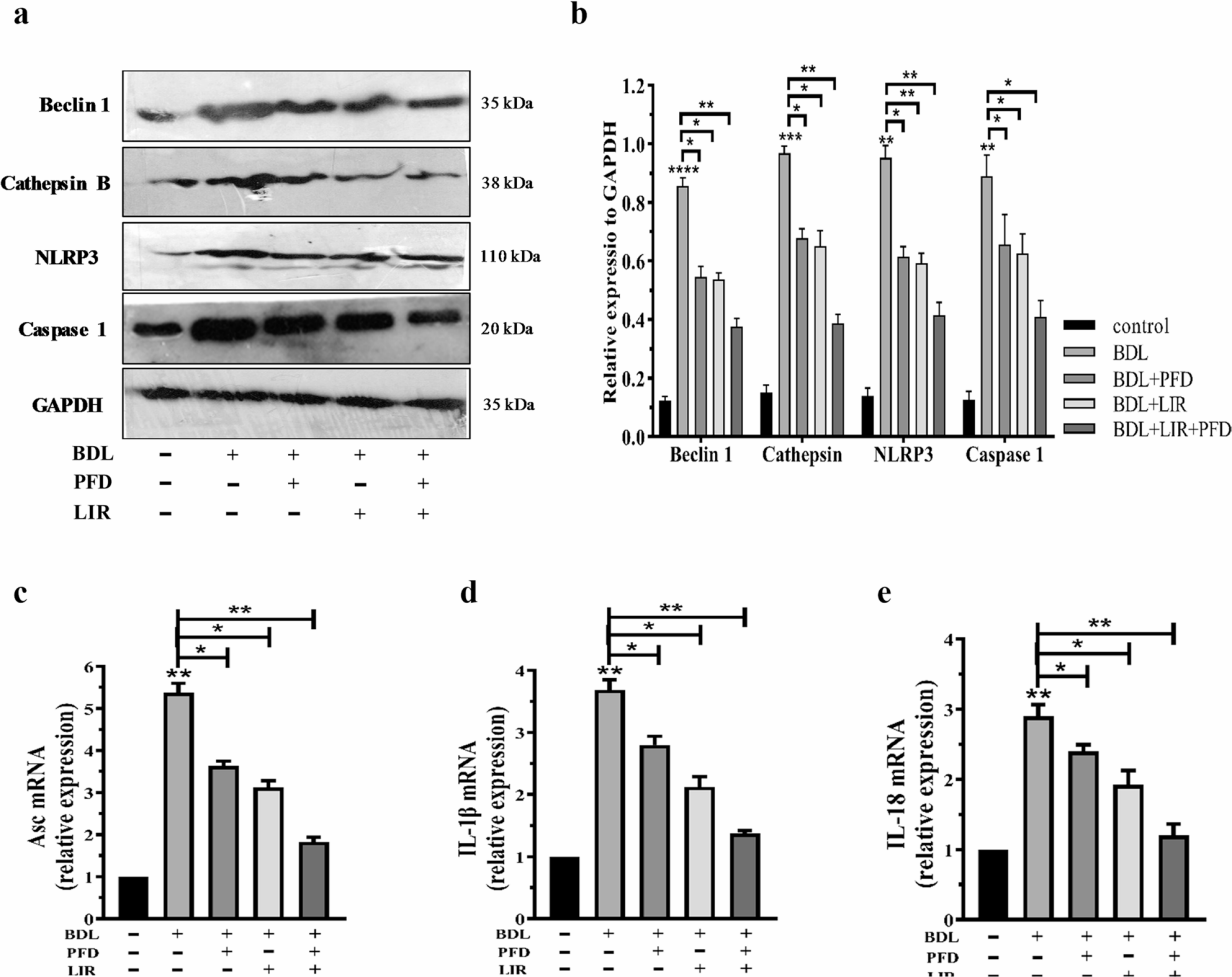
Effect of LIR and PFD alone and combination of LIR + PFD on the elevated levels of autophagy and NLRP3 inflammasome activation in the BDL-induced LF rats. **a** Representative protein expression of Beclin 1, Cathepsin B, NLRP3 and Caspase 1 in liver tissue using western blotting. The data were normalized to GAPDH levels. **b** Densitometric quantitation of Beclin 1, Cathepsin B, NLRP3 and Caspase 1. The mRNA levels of (**c**) Asc, (**d**) IL-1β and (**e**) IL-18. BDL: Bile duct ligation, PFD: Pirfenidone, LIR: Liraglutide, NLRP3: NOD-, LRR- and pyrin domain-containing protein 3, GAPDH: Glyceraldehyde 3-phosphate dehydrogenase, Asc: Apoptosis-associated speck-like protein containing a CARD, IL-1β: Interleukin-1β, IL-18: Interleukin-18. *p < 0.05, ** *p* < 0.01, *** *p* < 0.001, **** *p* < 0.0001

To further explore the mechanistic basis of the hepatoprotective effects, we examined markers of autophagy and NLRP3 inflammasome activation. The protein and mRNA levels of Beclin-1, NLRP3, Caspase-1, Asc, IL-1β, IL-18, and CTSB were analyzed by Western blotting and RT-PCR. The BDL group showed a significant increase in Beclin-1 expression, suggesting stress-induced autophagy associated with liver injury. Concurrently, there was marked upregulation of NLRP3, Caspase-1, IL-1β, IL-18, and CTSB, confirming robust inflammasome activation. Treatment with LIR or PFD alone significantly reduced Beclin-1 expression and downregulated inflammasome-related markers compared with the BDL group, indicating partial normalization of autophagy and inflammation (Fig. 5a & b). Notably, the LIR + PFD combination therapy was most effective in restoring Beclin-1 expression toward sham levels and markedly suppressing NLRP3, Caspase-1, and IL-1β/IL-18 activation. The combination also resulted in the strongest decrease in CTSB content, suggesting attenuation of lysosomal stress and inflammasome activation. Collectively, these results indicate that while both LIR and PFD modulate autophagy and inflammasome-related signaling markers, including NLRP3 and caspase-1 protein expression and IL-1β/IL-18 mRNA levels, suggesting suppression of NLRP3-associated inflammatory pathways (Fig. 5c). Their combined use more effectively normalizes autophagy flux and reduces NLRP3 inflammasome signaling indicator, contributing to the synergistic hepatoprotective effect observed in BDL-induced fibrosis.

### Combination therapy of LIR and PFD enhanced expression of proliferative and differentiation markers in rats with BDL-induced liver fibrosis

**Fig. 6:**
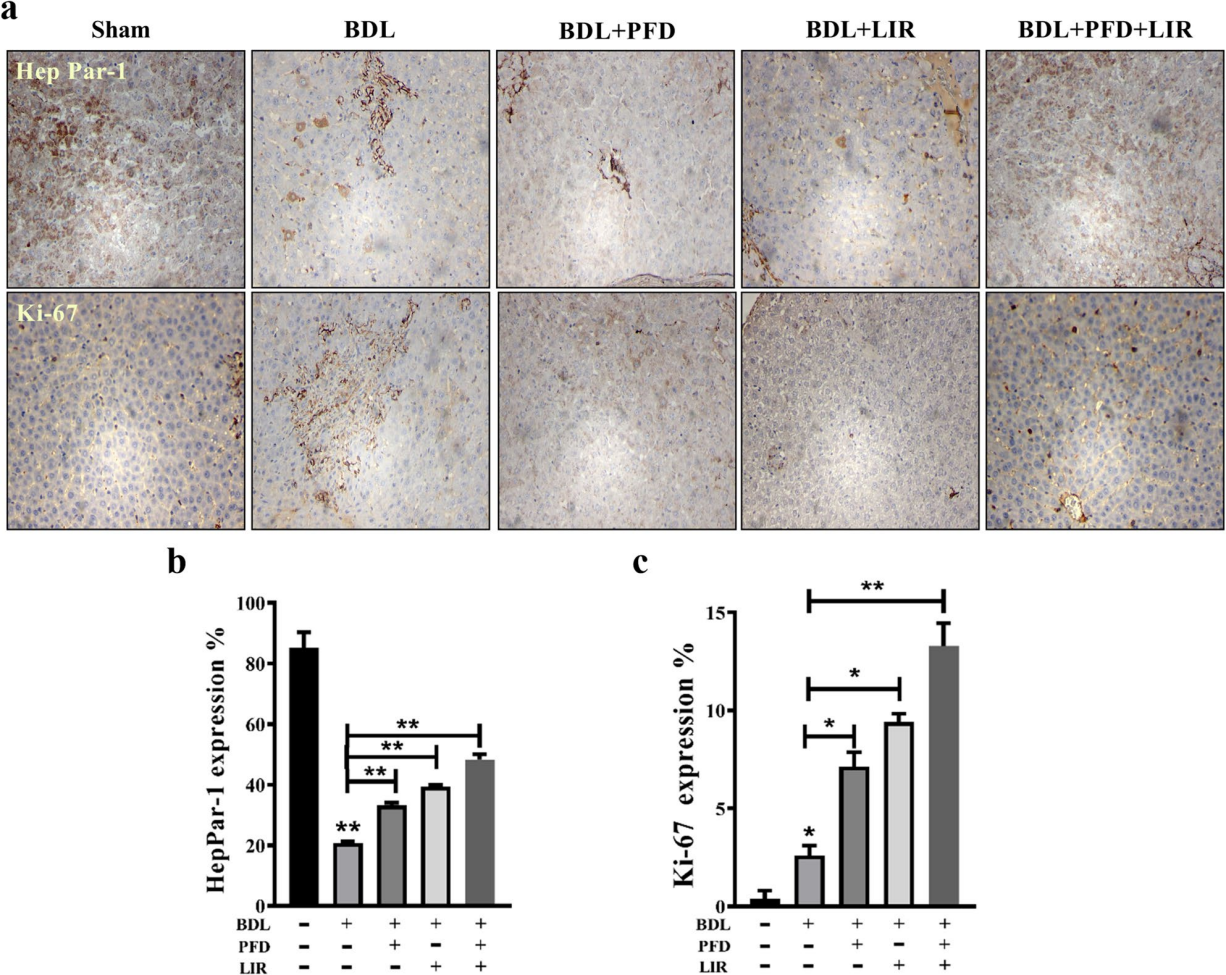
The effects of LIR, PFD, and their combination on HepPar-1 and Ki-67 expression were evaluated in liver sections from BDL-induced Wistar rats using immunohistochemistry. **a** Representative images of liver tissue stained for HepPar-1 and Ki-67 are shown, with a scale bar of 100 μm. Quantitative analysis involved counting the number of positive cells, and the results are expressed as the percentage of HepPar-1 and Ki-67 positive cells in each group (b and c). BDL: Bile duct ligation, PFD: Pirfenidone, LIR: Liraglutide, HepPar-1: Hepatocyte paraffin 1, Ki-67: marker of proliferation Kiel 67. *p < 0.05, ** *p* < 0.01

To evaluate the effects of PFD, LIR, and their combination on hepatocyte regeneration, we assessed HepPar-1 and Ki-67 expression using immunohistochemistry. As shown in Fig. 6a, the sham group exhibited strong, uniform, and granular cytoplasmic HepPar-1 staining, indicating normal hepatocyte function. The BDL group, however, showed markedly weakened and heterogeneous staining, reflecting impaired hepatocellular integrity and regeneration following bile duct ligation. Treatment with either LIR or PFD alone partially restored HepPar-1 immunoreactivity, showing moderate staining intensity relative to BDL. Notably, the combination therapy (LIR + PFD) resulted in a robust recovery of HepPar-1 expression, with dense and homogeneous staining similar to the sham group (Fig. 6b). In parallel, Ki-67 immunostaining (Fig. 6c) demonstrated a mild increase in nuclear positivity in the BDL group compared to sham, indicating limited compensatory expression of proliferative and differentiation markers. Both LIR and PFD monotherapies increased Ki-67 expression relative to BDL, while the LIR + PFD combination produced a significantly greater increase, suggesting enhanced hepatocyte proliferation and regenerative capacity. Together, these findings indicate that the combination therapy enhanced expression of proliferative and differentiation markers more effectively than either agent alone, likely through synergistic mechanisms that improve hepatocyte recovery following BDL-induced injury.

## Discussion

The present study demonstrates that combined administration of PFD and LIR exerts superior hepatoprotective and anti-fibrotic effects compared to either monotherapy in BDL-induced liver fibrosis. This combination therapy significantly improved liver function, reduced inflammation and ECM accumulation, normalized dysregulated autophagy markers, suppressed inflammasome indicators, and enhanced hepatocyte expression of proliferative and differentiation markers. To the best of our knowledge, this is the first study demonstrating that the PFD + LIR combination alleviates liver fibrosis by restoring the balance between autophagy and inflammasome pathways.

Hepatic fibrosis represents a common endpoint of various chronic liver diseases [1]. In the BDL model, the bile duct epithelium is the primary site of pathological damage, leading to severe cholestasis and progressive fibrosis. Although significant advances have been made in understanding the mechanisms of liver fibrosis and available treatments [40, 41], therapeutic options remain limited. Given the shortcomings of single-drug therapies, combination therapy emerges as a promising alternative approach [42, 43].

The synergistic effect of PFD and LIR may arise from their complementary actions on distinct hepatic cell populations and intersecting molecular pathways. LIR primarily acts on hepatocytes by activating GLP-1 receptors, improving cellular metabolism, and reducing oxidative stress, thereby indirectly attenuating hepatocyte-derived inflammatory signaling [44, 45]. In contrast, PFD mainly targets hepatic stellate cells (HSCs), inhibiting TGF-β–mediated activation and extracellular matrix (ECM) deposition [39, 46]. Despite acting on different cellular compartments, both agents converge on the suppression of the NLRP3 inflammasome, through distinct upstream mechanisms LIR via modulation of autophagy and mitochondrial ROS, and PFD via inhibition of profibrotic cytokine signaling [47, 48]. This dual targeting likely underlies the enhanced antifibrotic efficacy of their combination [49].

PFD, an anti-fibrotic agent clinically approved for idiopathic pulmonary fibrosis, has also shown beneficial effects in experimental liver fibrosis models [46]. Our previous work further demonstrated its hepatoprotective potential [39]. Considering the urgent need for effective anti-fibrotic agents [50], PFD-based combination strategies are being increasingly explored [51]. LIR, a GLP-1 receptor agonist, protects hepatocytes by preventing GLP-1 receptor internalization and reducing metabolic stress [44, 45]. Moreover, LIR has been reported to suppress NLRP3 inflammasome activation and decrease pro-inflammatory cytokine production [47, 48]. It also enhances autophagy-related proteins in a dose-dependent manner, suggesting that it may attenuate liver injury by modulating autophagic activity [49].

In our study, PFD + LIR treatment markedly improved serum liver enzyme levels, reduced the liver index, and restored hepatic histoarchitecture. Masson’s trichrome and Sirius Red staining revealed a significant reduction in collagen deposition relative to the BDL group. These histological improvements may result from reduced hydroxyproline content, normalization of liver index, and enhanced HepPar-1 expression. Consistent with prior reports [35, 52–56], both drugs alone exerted anti-fibrotic and anti-inflammatory effects, whereas their combination produced the most pronounced response. The reduction in NF-κB and TNF-α expression, alongside increased IL-10, confirmed the anti-inflammatory potential of the dual therapy. Additionally, elevated HepPar-1 and Ki-67 staining reflected enhanced hepatocyte proliferation and differentiation, suggesting improved regenerative capacity. The PFD + LIR combination also decreased mRNA expression of α-SMA, Col1a1, and Ccn2, supporting its role in suppressing key fibrogenic pathways.

In the BDL model, Beclin-1, an indicator of autophagy, was markedly upregulated, likely reflecting a maladaptive stress response. Excessive autophagy can lead to lysosomal destabilization and cathepsin B (CTSB) release, which subsequently activates the NLRP3 inflammasome and amplifies inflammation. We propose a mechanistic model whereby BDL-induced dysregulated autophagy triggers CTSB release, which in turn activates NLRP3, thereby promoting inflammation and fibrosis. The combined treatment with PFD and LIR restored autophagy to a protective, homeostatic level, interrupting this pathogenic feedback loop.

Although this study assessed NLRP3 and caspase-1 protein expression and IL-1β/IL-18 mRNA levels, definitive biochemical evidence of inflammasome activation—such as cleaved caspase-1 p20, mature IL-1β/IL-18 proteins, or ASC speck formation—was not obtained. These gold-standard markers will be incorporated in future investigations to further validate our findings.

Autophagy maintains cellular homeostasis by removing damaged organelles and misfolded proteins [57, 58]. However, it acts as a double-edged sword in liver injury, being either protective or detrimental depending on the context and duration of activation [59]. The marked Beclin-1 upregulation observed in the BDL group may reflect stress-induced or maladaptive autophagy, contributing to hepatocyte injury and inflammasome activation. While autophagy provides energy and promotes mitochondrial turnover, excessive or dysregulated autophagy can exacerbate cell death and inflammation. The PFD + LIR combination normalized Beclin-1 expression, reducing inflammation and fibrogenesis. Thus, restoration—not suppression—of autophagy appears to be a key mechanism underlying the therapeutic benefits of this combination.

Our findings showed that the PFD + LIR combination markedly reduced Beclin-1, suggesting that normalization of autophagy marker may decrease hepatocyte stress, inflammation, and necrosis. This interpretation aligns with the observed reductions in CTSB and NLRP3 inflammasome markers, supporting the notion that the therapy mitigates maladaptive autophagy and its downstream inflammatory consequences.

Previous studies indicate that autophagy and NLRP3 mutually regulate each other [60]. Macrophage autophagy influences inflammasome activation and thereby modulates the liver’s immune response [61]. Conversely, autophagy suppression leads to abnormal apoptosis and worsens hepatocyte dysfunction [62]. Functional autophagy protects the liver by preventing the accumulation of damaged mitochondria that release molecules activating inflammasomes [63, 64]. By maintaining autophagic flux, autophagy limits IL-1β and IL-18 production, reduces caspase-1 activation, and promotes NLRP3 degradation [65].

The lysosomal protease CTSB has also been implicated in inflammasome activation under conditions of autophagy dysregulation [66, 67]. In this study, BDL markedly increased hepatic CTSB, which may have triggered NLRP3 activation and pro-inflammatory cytokine release, accelerating fibrosis progression. PFD + LIR treatment significantly reduced CTSB expression, consistent with prior findings that CTSB release activates NLRP3 following liver injury [68]. Because excessive autophagy can destabilize lysosomes, reducing CTSB levels may help prevent NLRP3 activation and inflammation [69].

Beyond its anti-fibrotic and anti-inflammatory actions, the PFD + LIR combination promoted hepatocyte proliferation, as indicated by increased HepPar-1 and Ki-67 staining. This suggests that the combination not only prevents fibrosis progression but also facilitates hepatic regeneration by creating an anti-inflammatory, homeostatic environment conducive to repair.

Although this study provides valuable insights into the anti-fibrotic and hepatoprotective effects of the combination of pirfenidone and liraglutide, some limitations should be acknowledged. First, only male rats were used, and possible gender differences in liver fibrosis progression and drug metabolism were not evaluated. Second, the study evaluated outcomes at a single time point (20 days post-BDL), which may not reflect long-term therapeutic effects. Third, we did not perform a dose–response analysis for the combination therapy, and therefore the optimal therapeutic ratio of both drugs remains to be determined. Finally, while the BDL model is well established for studying cholestatic fibrosis, it may not fully represent other etiologies of liver fibrosis, such as metabolic or toxic injuries. Future studies addressing these limitations are warranted to better translate our findings to clinical settings. Additionally, we acknowledge that Ki-67 and HepPar-1 are general indicators of proliferation and hepatocellular differentiation, respectively, and may not fully distinguish hepatocyte regeneration from ductular or progenitor proliferation. Future studies using PCNA/BrdU incorporation or double immunostaining with hepatocyte- and cholangiocyte-specific markers could provide more definitive evidence of regeneration.

## Conclusion

**Fig. 7:**
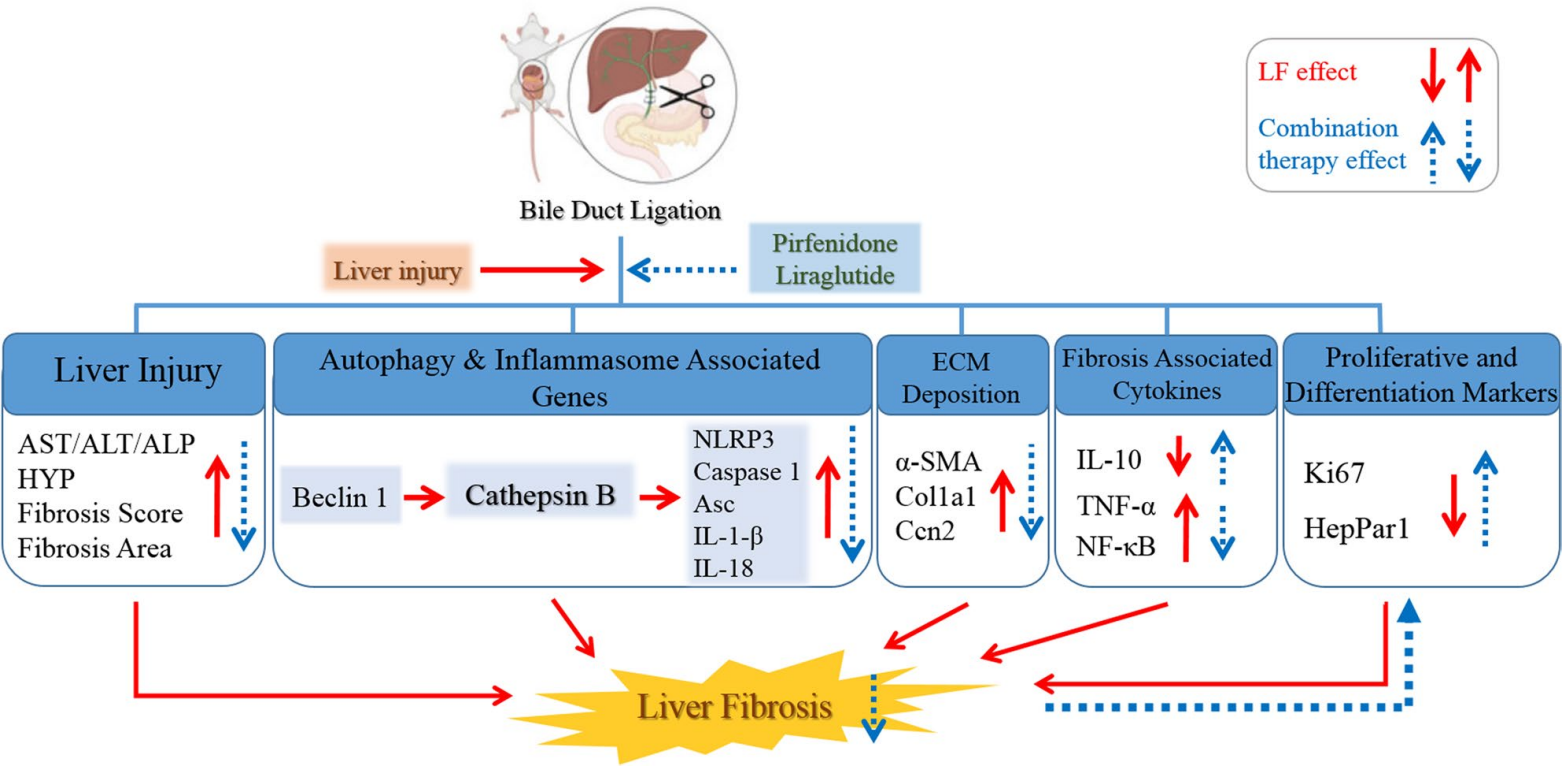
Theoretical schematic of LIR and PFD combination therapy in rats with BDL-induced liver fibrosis. BDL: Bile duct ligation, PFD: Pirfenidone, LIR: Liraglutide, ALT: Alanine aminotransferase; AST: Aspartate aminotransferase; ALP: alkaline phosphatase, HYP: Hydroxyproline, α-SMA: Alpha Smooth Muscle Actin, Col1a1: Collagen Type I Alpha 1, Ccn2: Cellular Communication Network Factor 2, TNF-α: Tumor Necrosis Factor alpha; NF-κB: Nuclear factor kappa-light-chain-enhancer of activated B, IL-10: Interleukin-10, NLRP3: NOD-, LRR- and pyrin domain-containing protein 3, GAPDH: Glyceraldehyde 3-phosphate dehydrogenase, Asc: Apoptosis-associated speck-like protein containing a CARD, IL-1β: Interleukin-1β, IL-18: Interleukin-18, HepPar-1: Hepatocyte paraffin 1, Ki-67: marker of proliferation Kiel 67

The current study provides compelling evidence that the combined administration of PFD and LIR enhances the regenerative response, normalizes Beclin-1 expression, and modulates the NLRP3 inflammasome pathway, thereby alleviating liver fibrosis (LF) (Fig. 7). Our findings offer new insights into the mechanisms underlying the release of pro-inflammatory cytokines associated with autophagy, demonstrating that autophagy-related markers are closely linked to NLRP3 inflammasome activation. Further investigations into the dynamic interplay between autophagy and inflammasome signaling during the progression of LF in hepatocytes and animal models would provide a deeper understanding of fibrosis pathophysiology and potential therapeutic interventions. Collectively, these findings position the PFD and LIR combination as a promising multi-target therapeutic strategy worthy of further clinical investigation for the treatment of liver fibrosis.

## Supplementary Information

Supplementary Material 1.

Supplementary Material 2.

## Data Availability

All data analyzed during this study are included in this published article and its supplementary information files. Additional details or materials are available from the corresponding author upon reasonable request.

## Abbreviations

LF: Liver fibrosis
PFD: Pirfenidone
LIR: Liraglutide
BDL: Bile Duct Ligation
ALT: Alanine aminotransferase
AST: Aspartate aminotransferase
ALP: Alkaline phosphatase
ECM: Extracellular Matrix
H&E: Haematoxylin and Eosin
HSC: Hepatic Stellate Cell
α-SMA: α-smooth muscle actin
MFB: Myofibroblast
TGF-β: Transforming Growth Factor β
ROS: Reactive Oxygen Species
HepPar-1: Hepatocyte Paraffin 1
Hyp: Hydroxyproline
TNF-α: Tumor Necrosis Factor-alpha
GAPDH: Glyceraldehyde 3-phosphate dehydrogenase
NF-κB: Nuclear Factor Kappa B
NLRP3: NOD-, LRR- and pyrin domain-containing protein 3
CTSB: Cathepsin B
