# Supplementary material for: Protective anti-fibrotic effect of liraglutide and Pirfenidone combination therapy on liver fibrosis in rats: effects on autophagy and NLRP3 inflammasome
Source: BMC Gastroenterol. 2025 Dec 18;26:57. doi: 10.1186/s12876-025-04545-z (PMC12831306; doi:10.1186/s12876-025-04545-z)

cathepsin

**Fig 5 a.** Beclin1- 35 kDa


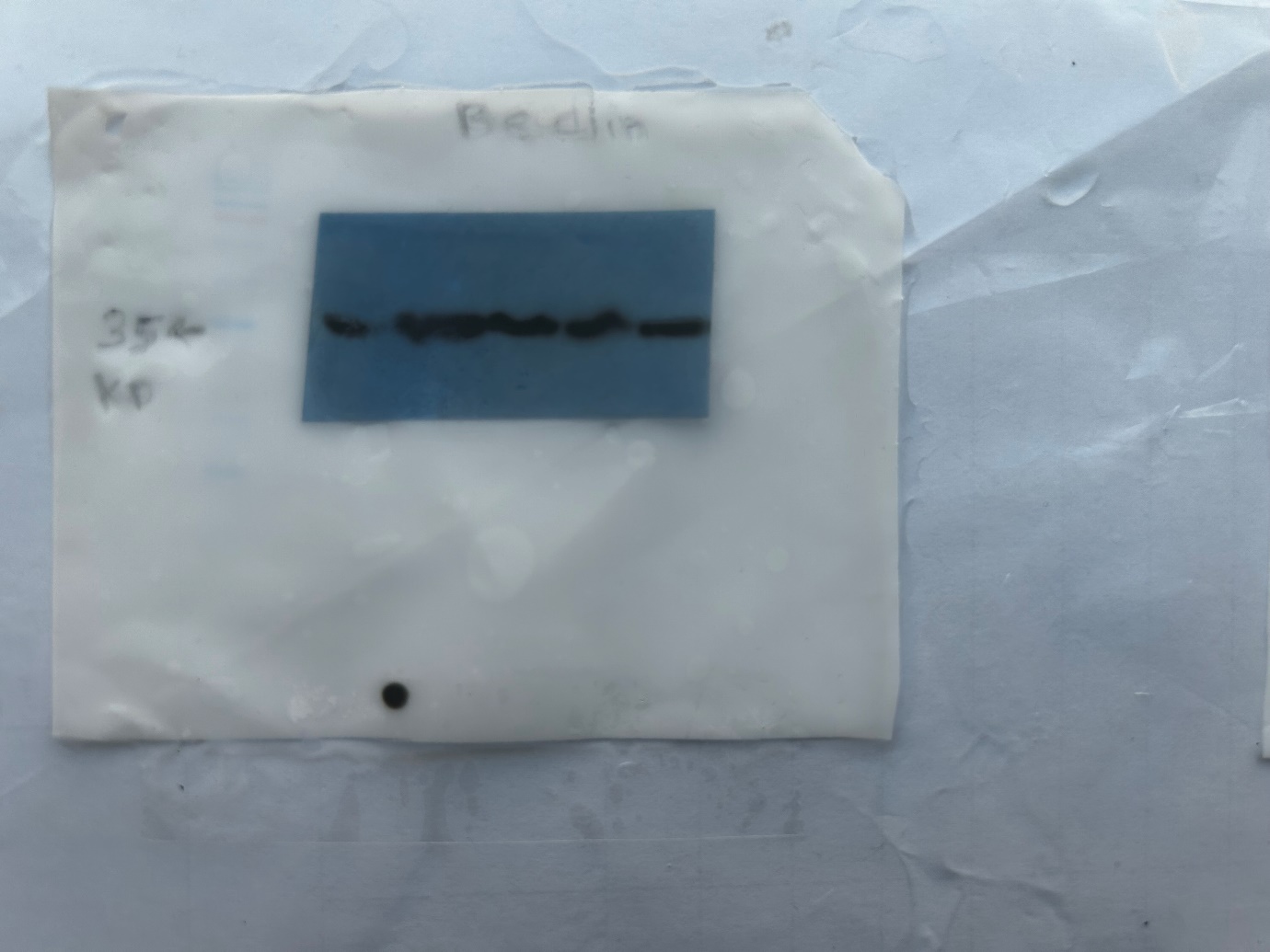


**Fig 5 a.** Cathepsin B- 38 kDa


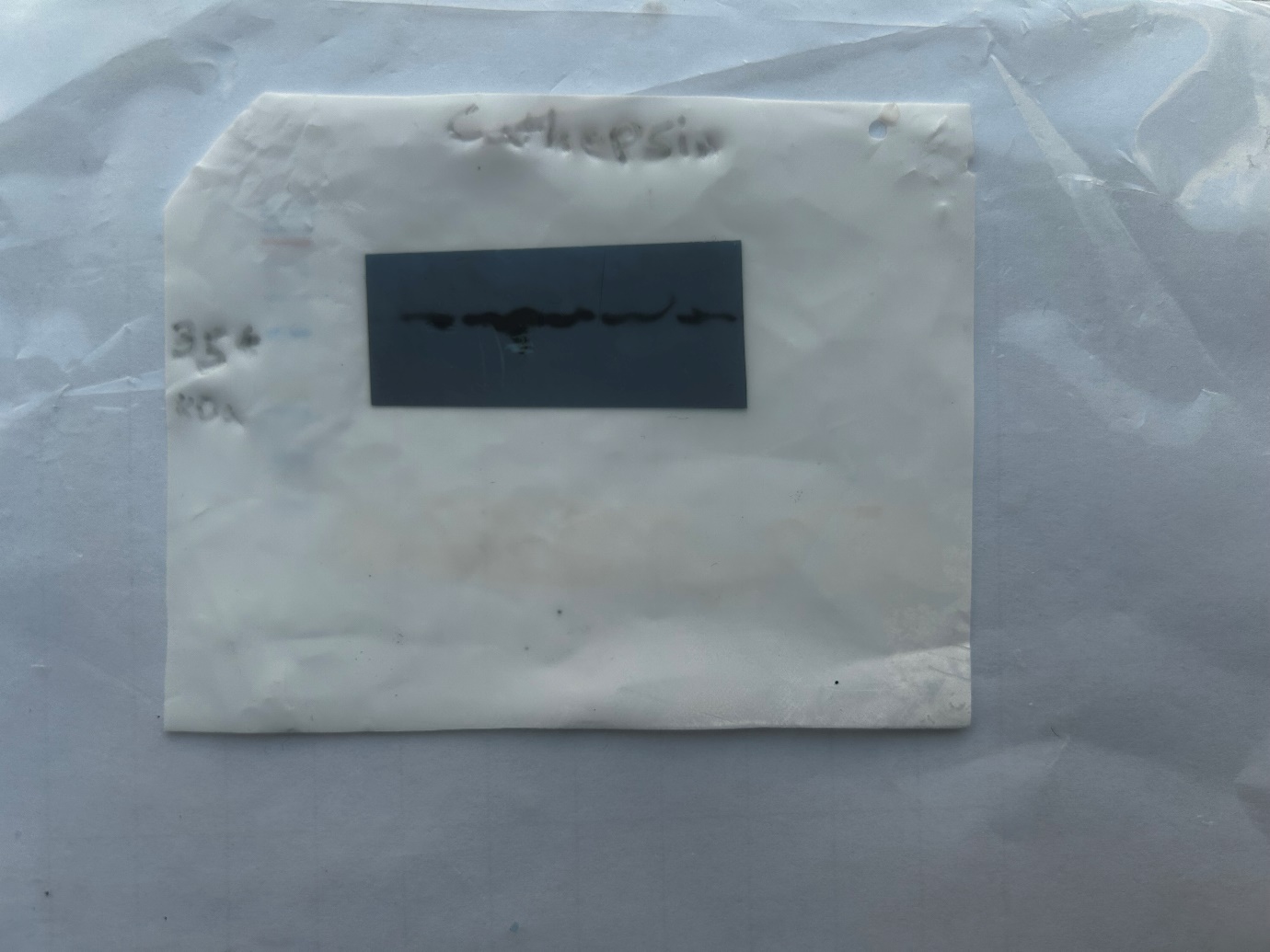


**Fig 5 a.** NLRP-3-110 kDa


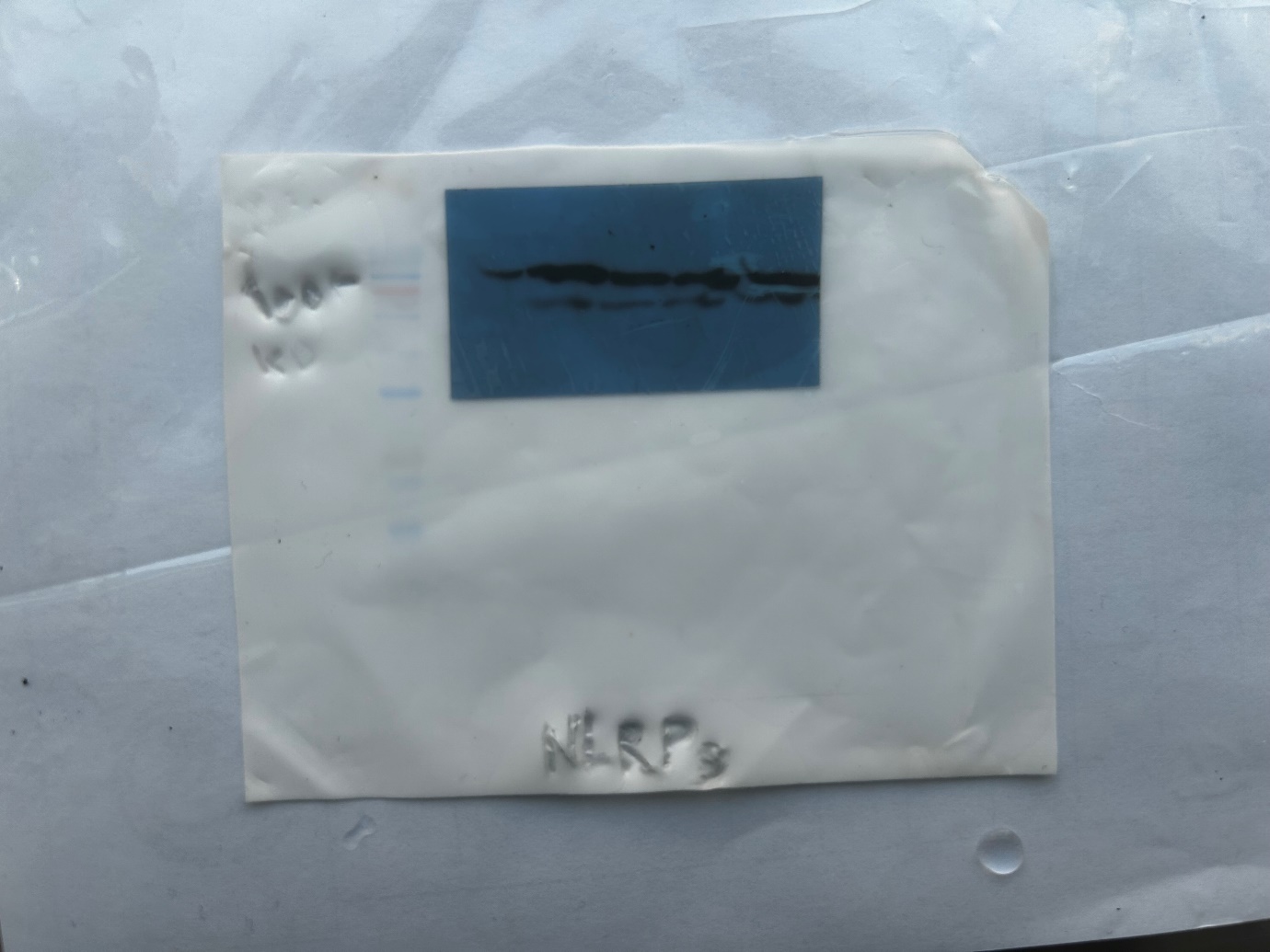


**Fig 5 a.** Caspase 1- 20 kDa


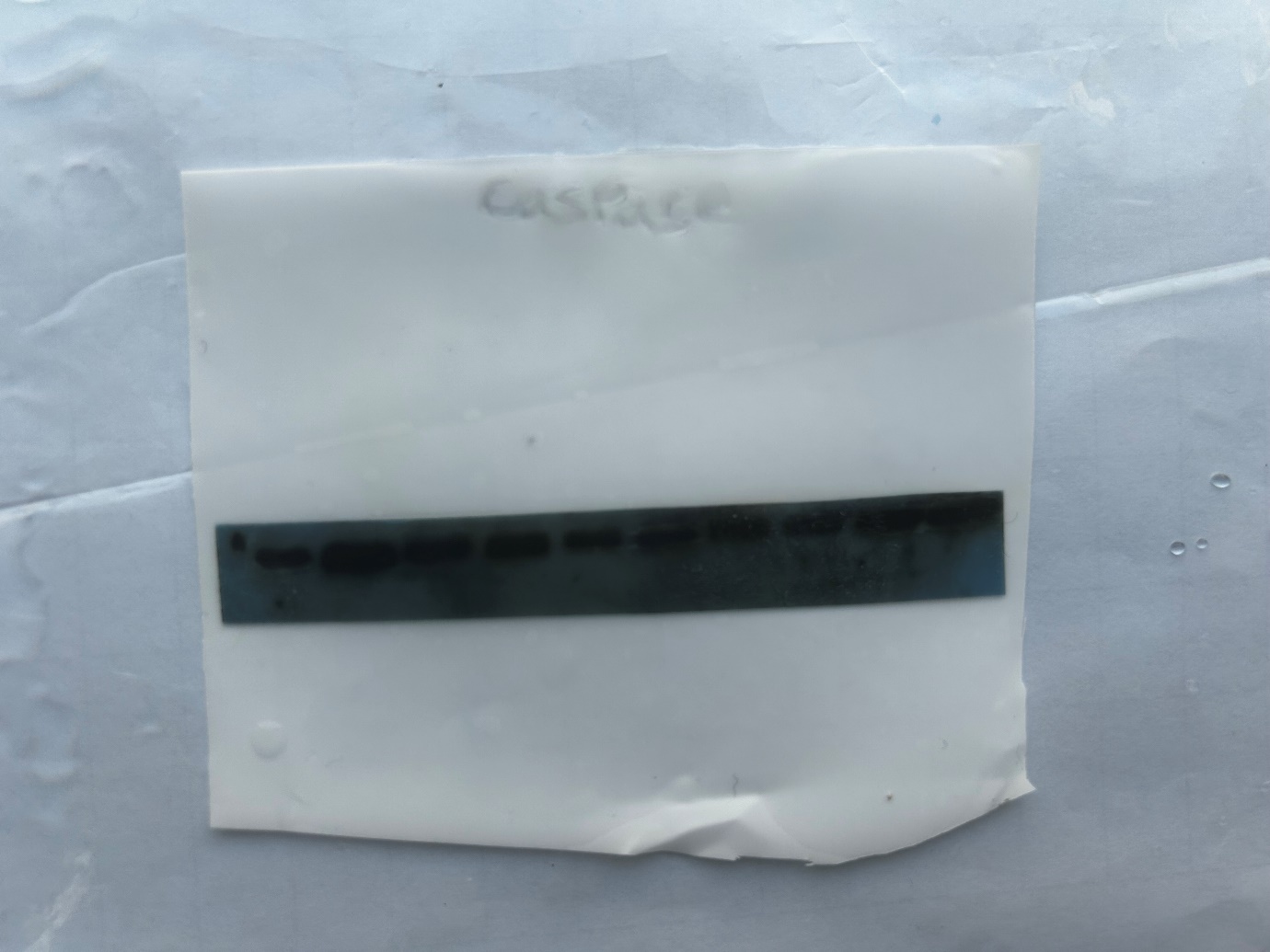


**Fig 5 a.** GAPDH- 35 kDa


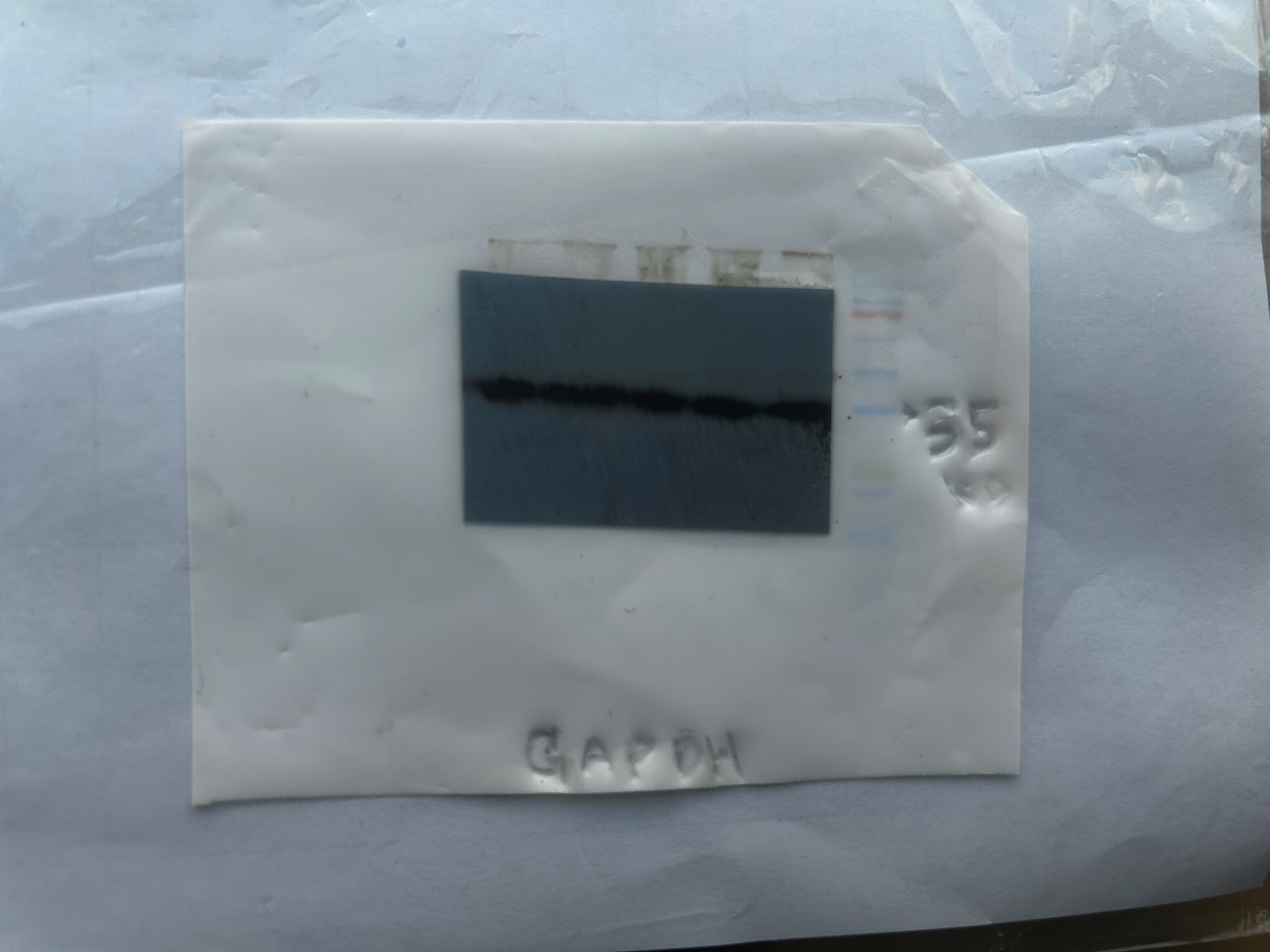


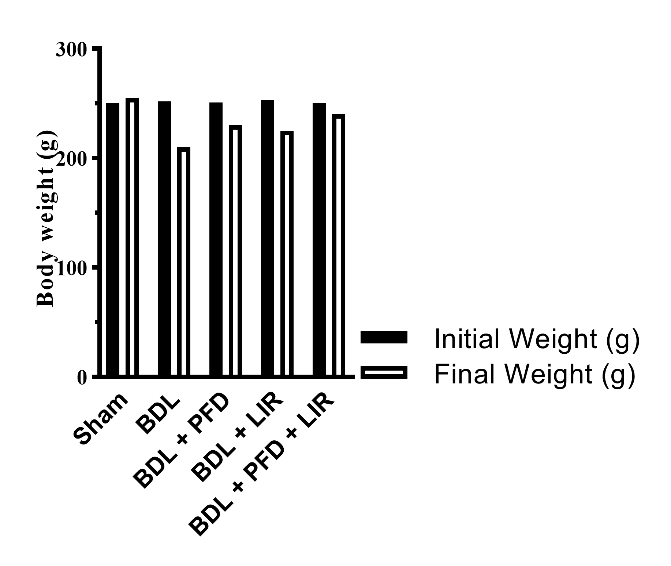


**Figure S2.** Changes in body weight from the start to the end of the experiment in each group. Data are presented as mean ± SD (n = 10 per group).

No significant differences in initial body weights were observed among groups. The final body weights showed only minor, non-significant changes, indicating that the hepatoprotective effects were not primarily driven by liraglutide-induced weight loss.”


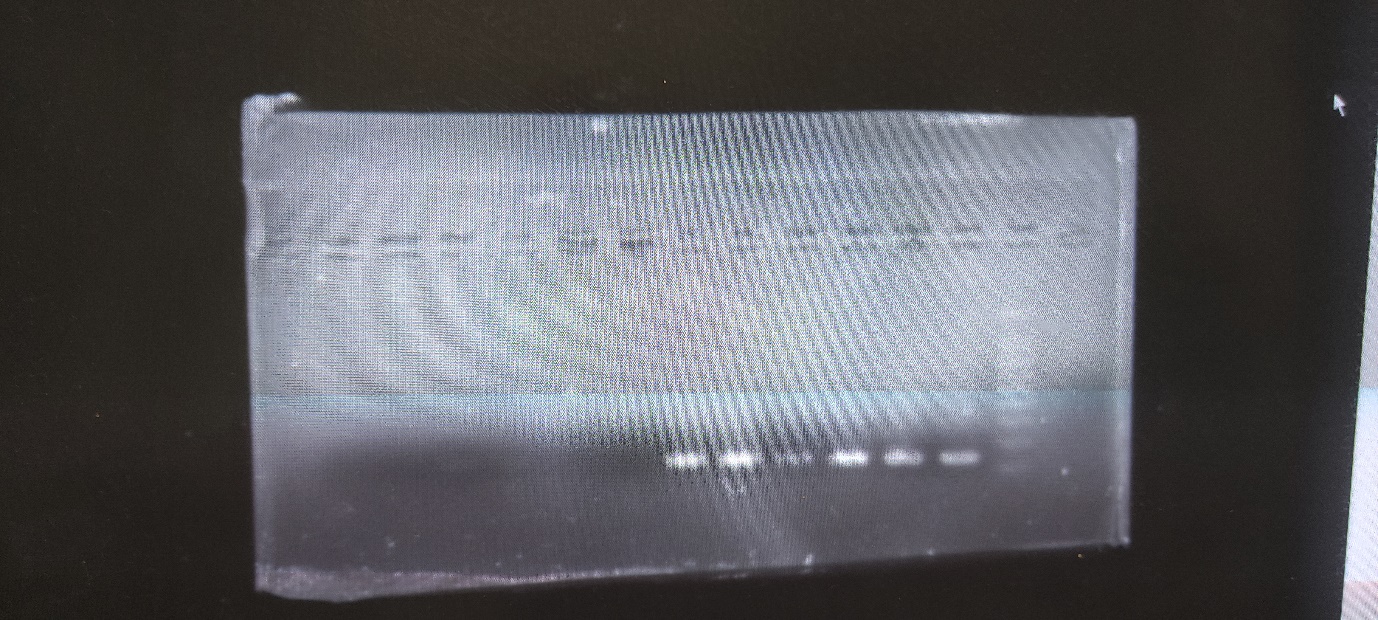


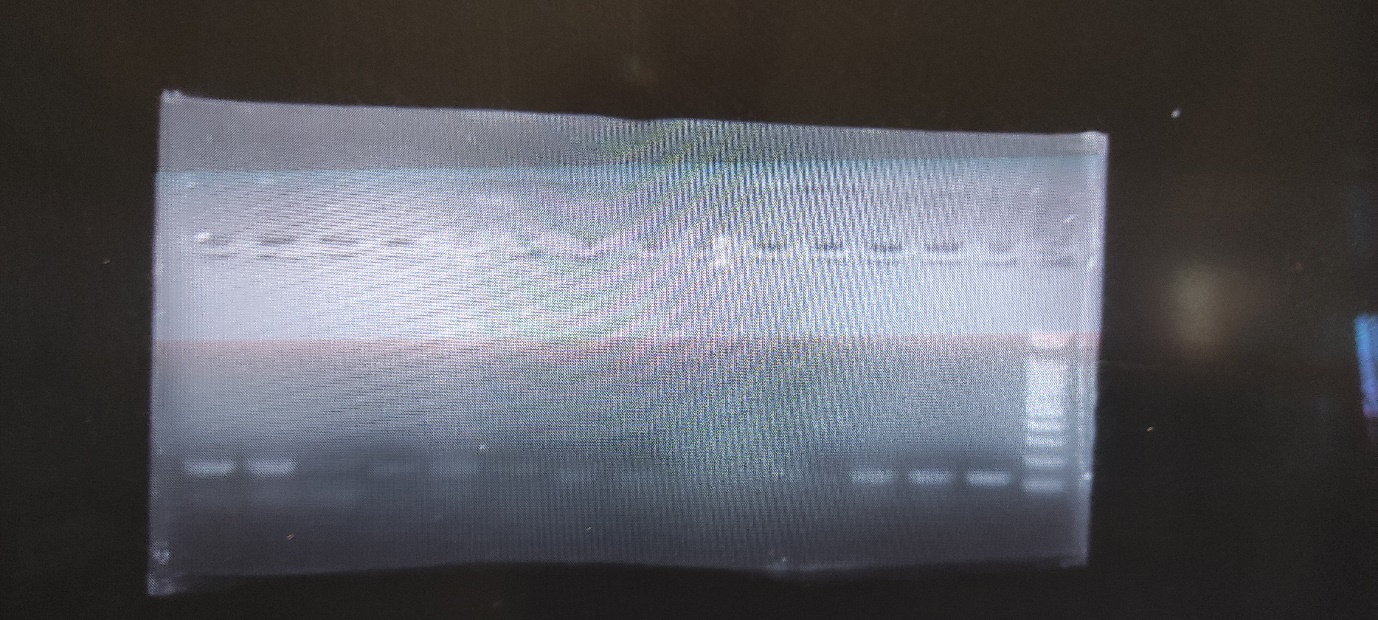


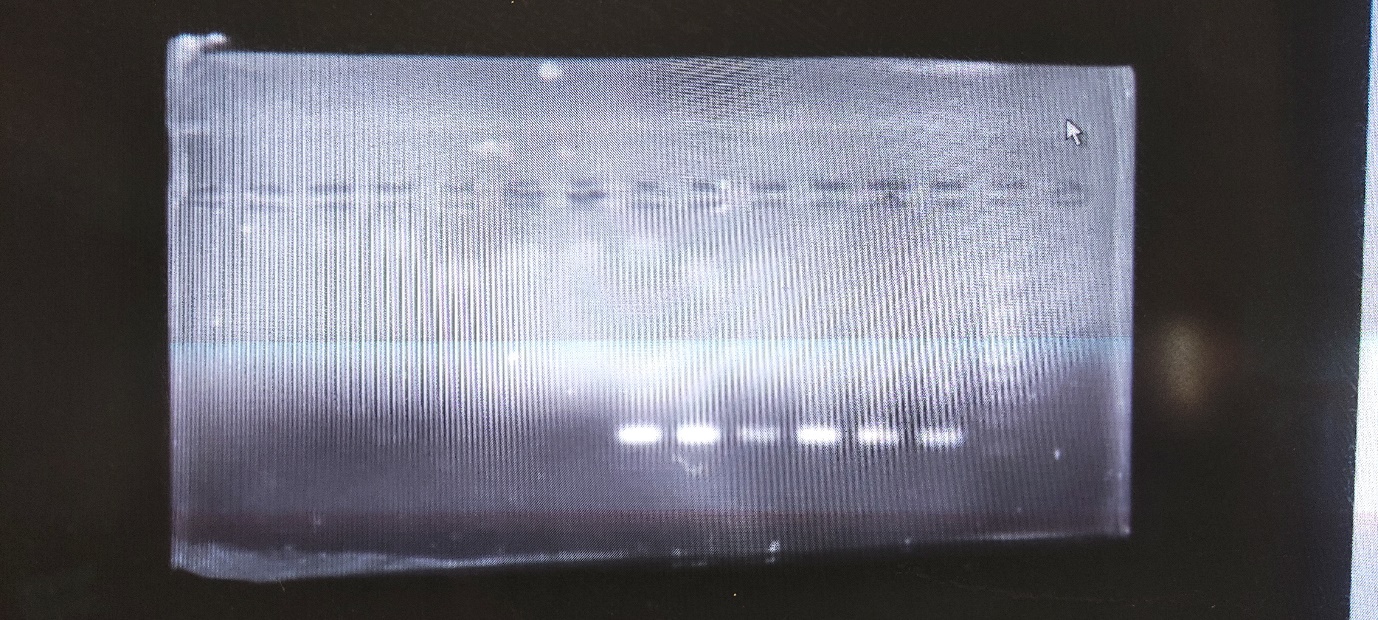


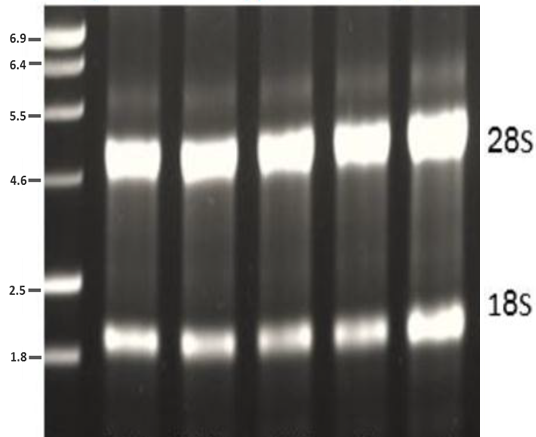


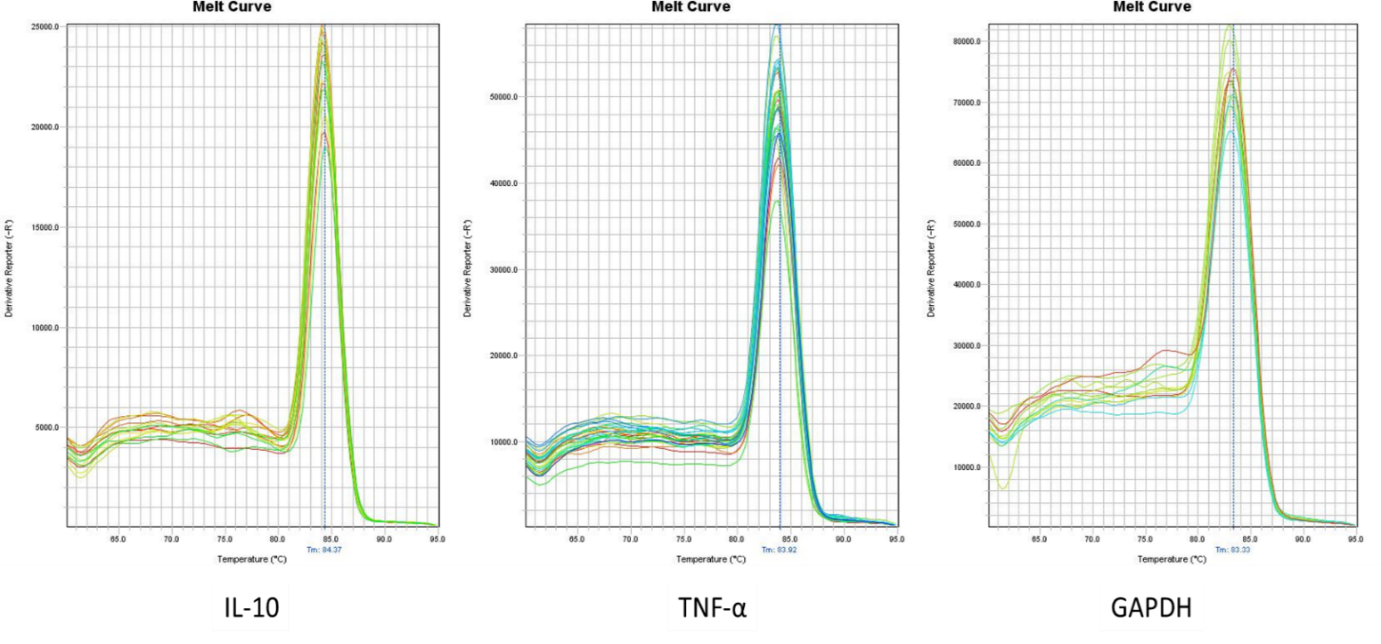


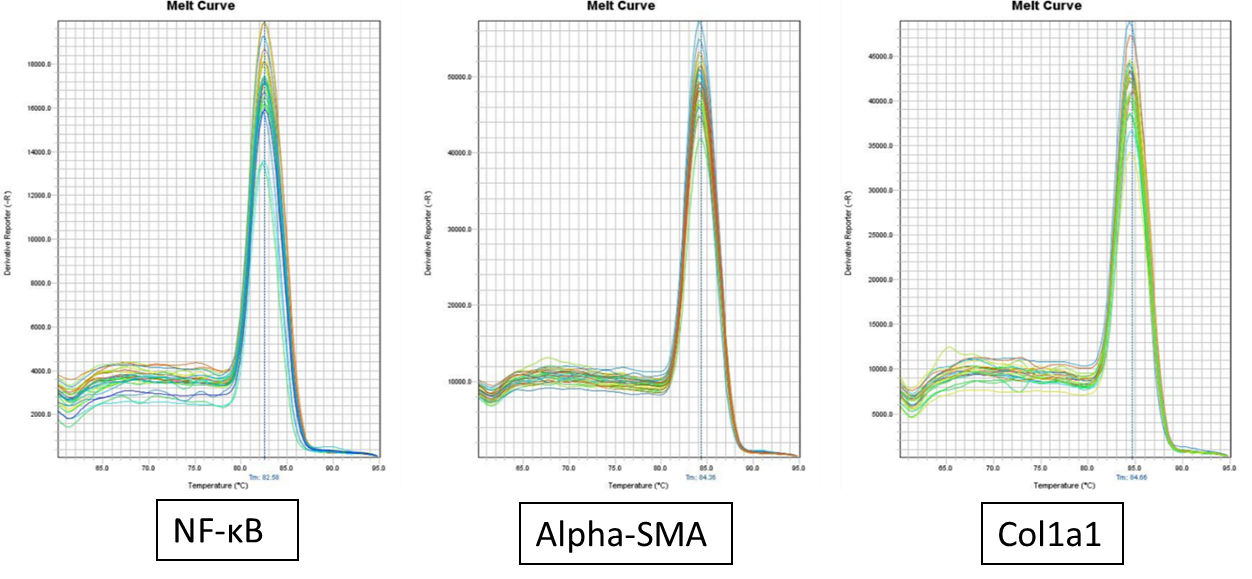

Supplement: Supplementary file 1 — Supplementary Material 1. [file 12876_2025_4545_MOESM1_ESM.docx]
