# Supplementary material for: Protective anti-fibrotic effect of liraglutide and Pirfenidone combination therapy on liver fibrosis in rats: effects on autophagy and NLRP3 inflammasome
Source: BMC Gastroenterol. 2025 Dec 18;26:57. doi: 10.1186/s12876-025-04545-z (PMC12831306; doi:10.1186/s12876-025-04545-z)
Supplement: Supplementary file 2 — Supplementary Material 2. [file 12876_2025_4545_MOESM2_ESM.docx]

| Target Gene | Primer sequences (Forward and Reverse) | |
| --- | --- | --- |
| Il-18 | *Forward* | 5'-GGCTCTTGTGTCAACTTCAAA-3' |
|  | *Reverse* | 5'-TTATCAGTCTGGTCTGGGATT-3' |
| Asc | *Forward* | 5'- GCTCACAATGTCTGTGCTTAGAG-3' |
|  | *Reverse* | 5'- GCAGTAGCCACAGCTCCAG-3' |
| *TNF-α* | *Forward* | 5'-TGAACTTCGGGGTGATCGGTC-3' |
|  | *Reverse* | 5'-TTGGTGGTTTGCTACGACGTG-3' |
| *IL-10* | *Forward* | 5'- TCCACTTCCCAGTCAGCCAG -3' |
|  | *Reverse* | 5'- TCACCCAAGTAACCCTTAAAGTCC -3' |
| *GAPDH* | *Forward* | 5'-TCATCAACGGCACAGTCAAGG-3' |
|  | *Reverse* | 5'-TTCTGCATGGTGGTGAAGACG-3' |
| IL-1 | *Forward* | 5'-TGAGCTTTCGACAGTGAGGAG-3' |
|  | *Reverse* | 5'-TAGTCGAGATGCTGCTGTGAG-3' |
| NF-κB | *Forward* | 5'-CAGGGCATCCAGACCAACAA-3' |
|  | *Reverse* | 5'-TCGGCTTGAGAAAAGGAGCC-3' |
| α-SMA | *Forward* | 5'-GGAGATGGCGAGACTCACAA-3' |
|  | *Reverse* | 5'-GCTCAGCAGTAGTCACGAA-3' |
| Col1a1 | *Forward* | 5'-CCCAGCCGCAAAGAGTCTAC-3' |
|  | *Reverse* | 5'-CAGGTTTCCACGTCTCACCA-3' |
| Ccn2 | *Forward* | 5'-ATGGTCCACCCTGTGTCTTC-3' |
|  | *Reverse* | 5'-ACTCCTCACAGCATTTCCC-3' |
